# Progress in molecular markers associated with radiotherapy efficacy in glioma

**DOI:** 10.3389/fonc.2026.1734404

**Published:** 2026-04-27

**Authors:** Xiang Gao, Yijing Ren, Lishan Gao, Hao Yu, Ping Zhou

**Affiliations:** 1 Department of Geratology, Nanhu District People’s Hospital, Jiaxing, China; 2 The First Affiliated Hospital of Hainan Medical University, The First School of Clinical Medicine, Haikou, China; 3 Department of Ophthalmology, Haining People’s Hospital, Jiaxing, China; 4 Institute of Biomedical and Health Engineering, Shenzhen Institutes of Advanced Technology, Chinese Academy of Sciences, Shenzhen, China; 5 The Key Laboratory of Biomedical Imaging Science and System, Chinese Academy of Sciences, Shenzhen, China; 6 Department of Biomedical Engineering, Shenzhen University of Advanced Technology, Shenzhen, China; 7 Radiotherapy Department II, Key Laboratory of Emergency and Trauma of Ministry of Education, The First Affiliated Hospital, Hainan Medical University, Haikou, China

**Keywords:** AI-driven frameworks, gliomas, mechanisms, molecular markers, radiotherapy efficacy

## Abstract

Radiotherapy remains a cornerstone in glioma treatment, yet its efficacy is significantly hindered by tumor heterogeneity and molecularly driven radioresistance. This review systematically delineates molecular biomarkers that influence radiotherapy outcomes, categorizing them into radiosensitivity (e.g., IDH1 mutations, MGMT promoter methylation, TIM-3) and radioresistance (e.g., CD133, CD44, PRMT1, CSF-1R,RAD51,HMGB2). Mechanistically, radiosensitivity is governed by DNA repair fidelity (MGMT), ferroptosis suppression (PRMT1), and immune modulation (TIM-3/TAMs). Radioresistance arises from cancer stem cell maintenance (CD133/HMGB2), TAM polarization (CSF-1R/CD44), and enhanced homologous recombination (RAD51). Integrating molecular stratification into radiotherapy paradigms demonstrates clinical utility: MGMT methylation permits radiation dose de-escalation (52–54 Gy vs. 60 Gy) without compromising survival (32 vs. 25 months), while TIM-3 expression predicts responsiveness to combinatorial immunotherapy. A multi-omics AI model combining radiomics, dosiomics, and clinical data to predict radiotherapy response in glioma. Using a support vector machine trained on 176 patients, the fused model achieved an AUC of 0.728(95% CI:0.717–0.739) in validation, outperforming single-modality approaches. These advances underscore the transformative potential of biomarker-guided precision radiotherapy, enabling tailored interventions that counteract resistance mechanisms and synergize with immunotherapies. By bridging molecular insights with clinical innovation, this paradigm shift promises to redefine glioma management, offering renewed hope for overcoming therapeutic recalcitrance in this devastating malignancy.

## 1 Introduction

Gliomas represent the most prevalent and aggressively malignant primary brain tumors, characterized by rapid progression, pervasive recurrence, and dismal prognoses (1). Glioblastoma (GBM), the prototypical example, demonstrates a 5-year survival rate of merely 7.2%, with median overall survival (OS) of 9.8–10 months following standard therapy (2). Local recurrence remains highly prevalent and is generally associated with poor survival outcomes, reflecting the substantial therapeutic resistance characteristic of recurrent disease. Current therapeutic paradigms emphasize maximal safe resection followed by adjuvant chemoradiation, wherein radiotherapy maintains indispensable clinical utility (3). Cohort analyses reveal superior outcomes with concurrent chemoradiation: median OS extends to 13.5 months (95% CI: 10.9–16.6) with 21.7% 2-year survival, while gross total resection (GTR) recipients achieve exceptional 58.5-month median OS versus 13.2 months in subtotal resection cohorts (p=0.0000426), unequivocally underscoring radiotherapy’s centrality in multimodal management (4). Nevertheless, therapeutic efficacy remains constrained by intrinsic radioresistance mechanisms, molecular heterogeneity, and immunosuppressive tumor microenvironmental dynamics. Post-radiotherapy recurrence rates persist at 80%, necessitating urgent development of optimized therapeutic paradigms integrating molecular stratification and microenvironmental modulation to transcend current therapeutic plateaus (5).

The conventional glioma classification system—namely the WHO Central Nervous System (CNS) grading framework—relies primarily on histopathological features and exhibits inherent limitations in guiding therapeutic decision-making, particularly due to its inability to account for intragrade heterogeneity in radiosensitivity (6). This morphology-based paradigm fails to reconcile the paradox of widely divergent clinical outcomes among glioblastoma patients, all uniformly classified as WHO grade IV, who nevertheless experience progression-free survival ranging from 3 to 28 months under identical radiotherapy protocols (p < 0.001) (7). In contrast, molecular stratification introduces a paradigm shift by employing functionally relevant biomarkers. Diagnostic classifiers such as IDH1/2 mutations (achieving >95% accuracy in distinguishing primary from secondary glioblastomas) and 1p/19q co-deletion (pathognomonic for oligodendroglioma) offer enhanced diagnostic specificity (8). Prognostic markers, including TERT promoter mutations, are associated with significantly reduced median survival (19.6 vs. 10.2 months; HR = 2.1), while predictive biomarkers—most notably MGMT promoter methylation—triple the likelihood of temozolomide responsiveness in methylated cohorts (9). The updated 2021 WHO CNS tumor classification reflects a paradigm shift toward molecularly informed glioma classification, in which tumor entities are defined by key genetic alterations rather than histology alone (10). Despite these advances, several critical challenges remain. Univariate biomarker models continue to yield limited predictive accuracy, and efforts toward multi-omics integration remain largely experimental and fragmented. Furthermore, radiation oncology protocols have yet to adopt molecularly guided personalization. As a result, approximately 35% of patients with otherwise favorable molecular profiles fail to achieve expected survival outcomes—underscoring the urgent need for artificial intelligence–driven clinical decision-making frameworks that dynamically integrate molecular signatures with dosimetric optimization to enable truly individualized radiotherapy (11).

**Figure 1:**
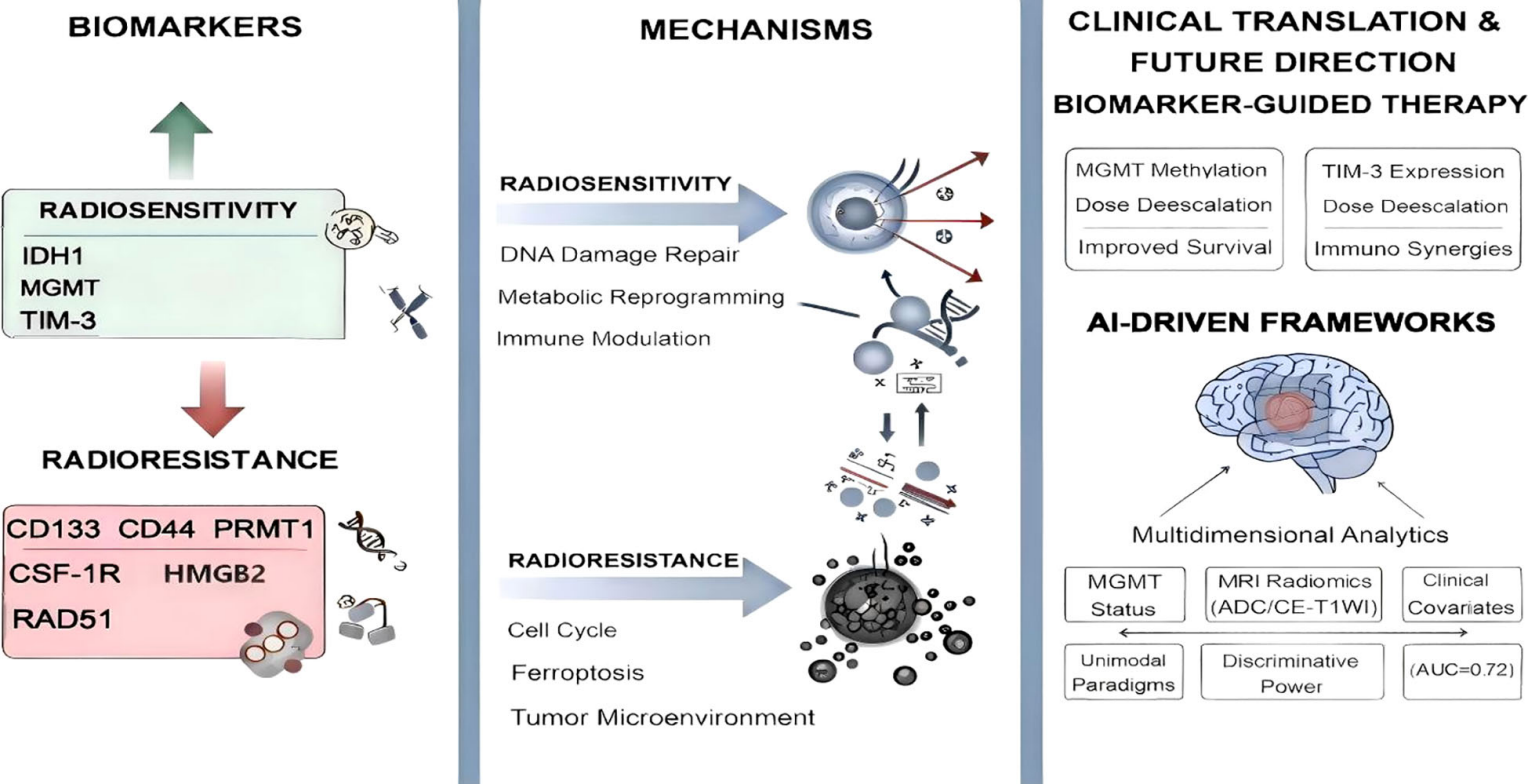
Biomarkers, mechanisms, and personalized radiotherapy.

This review aims to provide a comprehensive overview of the molecular markers of gliomas affecting radiotherapy. By elucidating the roles and mechanism of both positive and negative markers, we aim to highlight the potential for integrating molecular profiling into clinical practice to optimize radiotherapeutic interventions and thus improve glioma patient prognoses (**Figure 1**). Throughout this manuscript, radioresistance is defined as the capacity of tumor cells to withstand radiation-induced damage and continue to survive and proliferate, whereas radiosensitivity denotes the degree to which tumor cells are vulnerable to radiation-mediated cytotoxic effects.

## 2 Radiosensitivity molecular markers

### 2.1 IDH1

IDH1 (Isocitrate Dehydrogenase 1) is located on chromosome 2q33 and encodes the enzyme isocitrate dehydrogenase 1 (12). IDH1 mutations are extensively recognized as potent predictive markers for the efficacy of radiotherapy in glioma patients. A retrospective clinical study indicates that tumors with IDH1 mutations demonstrate superior responses to radiotherapy, manifesting more pronounced reductions in tumor volume and significantly improved outcomes when combined with chemotherapy (13–15). For instance, in patients undergoing concurrent radiochemotherapy, IDH1 mutations correlate with a substantial extension in progression-free survival (PFS) from 3.3 years to 8.4 years and an increase in OS from 4.5 years to 16.3 years (**Table 1**) (16). These findings affirm IDH1 mutations as critical prognostic markers for radiotherapy efficacy.

**Table 1:** Radiotherapy efficacy molecular markers.

Biomarker	Radiotherapy efficacy	Evidence level	Reference
IDH1 MGMT TIM-3 CD133 CD44 PRMT1 CSF-1R RAD51 HMGB2	Radiosensitivity Radiosensitivity Radiosensitivity Radioresistance Radioresistance Radioresistance Radioresistance Radioresistance Radioresistance	Retrospective clinical study Retrospective cohort study Clinical cohort+Preclinical (*in vitro*) Retrospective clinical study Retrospective clinical study Preclinical (*in vitro*) Clinical trials + Preclinical (*in vitro*) Retrospective clinical study Preclinical (*in vitro*)	(16) (21, 22) (26, 27) (32, 33) (39) (41, 42) (44, 45) (53) (57)

IDH1—Isocitrate Dehydrogenase 1;MGMT—O-6-methylguanine-DNA methyltransferase;TIM-3—T-cell Immunoglobulin and Mucin-domain containing-3;CD133—Cluster of Differentiation 133;CD44—Cluster of Differentiation 44;PRMT1—Protein Arginine Methyltransferase 1;CSF-1R—Colony Stimulating Factor 1 Receptor;RAD51—RAD51 Recombinase;HMGB2—High Mobility Group Box Protein 2.

### 2.2 MGMT

MGMT (O6-Methylguanine-DNA Methyltransferase) is a DNA repair protein that maintains genomic stability by repairing alkylation-induced DNA damage (17). MGMT promoter methylation is and also widely acknowledged as a Radiosensitivity marker in glioma patients. Methylation impedes MGMT protein expression, thereby reducing DNA repair capacity and enhancing tumor cell sensitivity to radiotherapy and alkylating agents such as temozolomide (TMZ) (18–20). A retrospective cohort study demonstrate that patients with MGMT methylation have a significantly prolonged median OS of 21.7 months compared to 15.3 months in unmethylated patients. Additionally, median PFS extends to 10.3 months versus 5.9 months (p < 0.01) (21). In elderly cohorts, methylation status similarly predicts better therapeutic outcomes, with median OS reaching 12.7 months, markedly higher than the 8.2 months observed in unmethylated patients (p < 0.05) (22). Consequently, MGMT promoter methylation serves as a robust prognostic biomarker for radiotherapy efficacy in gliomas, providing critical guidance for individualized therapeutic strategies (**Table 1**).

### 2.3 TIM-3

TIM-3 (T-cell immunoglobulin and mucin-domain containing molecule-3), a transmembrane immune checkpoint protein, is broadly expressed on immune cells, including T cells, natural killer (NK) cells, and tumor-associated macrophages (TAMs) (23). Mechanistically, TIM-3 suppresses effector T-cell activation and drives T-cell exhaustion by binding ligands such as galectin-9 and phosphatidylserine, thereby fostering immune tolerance and tumor immune evasion (24). In gliomas, particularly glioblastoma (GBM), TIM-3 overexpression is driven by epigenetic dysregulation, specifically promoter hypomethylation, which correlates with advanced tumor malignancy and the establishment of an immunosuppressive tumor microenvironment (25). Clinical cohort analyses reveal that high TIM-3 expression in GBM is associated with significantly reduced median overall survival (OS) (11.3 months vs. 18.7 months in low expressers; p = 0.004), underscoring its prognostic relevance (26). Preclinical studies further demonstrate that TIM-3 blockade synergizes with radiotherapy, extending median survival in tumor-bearing mice from 17 to 25 days (p = 0.002) and yielding a 20% long-term survival rate. Remarkably, combining TIM-3 inhibition with anti-PD-1 therapy and stereotactic radiotherapy achieves complete survival (100%; p < 0.05) (27). These findings position TIM-3 as a promising therapeutic target in glioma treatment, offering a dual mechanism to counteract immunosuppression and enhance radiosensitivity, with significant translational implications for clinical practice (**Table 1**).

## 3 Radioresistance molecular markers

### 3.1 CD133

CD133, also known as Prominin-1, is a transmembrane glycoprotein extensively recognized as a marker for glioma cancer stem cells (GSCs) (28). High expression of

CD133 in tumor cells is closely associated with self-renewal capacity, invasiveness, and therapeutic resistance. The expression of CD133 is intrinsically linked to the stem-like properties of tumors, including resistance to radiotherapy and chemotherapy, thereby adversely affecting patient prognosis (29). Research indicates that glioma cells with elevated CD133 expression exhibit reduced sensitivity to radiotherapy and chemotherapy, primarily due to enhanced DNA damage repair capabilities and anti-apoptotic characteristics (30). Additionally, the proportion of CD133-positive cells significantly increases post-radiotherapy, as these cells demonstrate superior proficiency in repairing radiation-induced DNA damage, leading to augmented radiotherapy resistance and tumor recurrence (31).Empirical data reveal that recurrence after radiotherapy GBM patients with high CD133 expression have a markedly shorter median OS of 16 months compared to 40 months in low-expression patients (p = 0.04) (32). Furthermore, a retrospective clinical study elevated CD133 expression is significantly associated with reduced progression-free survival (PFS) (HR = 2.03, 95% CI: 1.43–2.88, p < 0.001) (33). Therefore, CD133 gene alterations serve as negative prognostic predictors for radiotherapy efficacy and overall prognosis, suggesting that CD133-targeted therapies may represent a crucial strategy to enhance radiotherapeutic outcomes in gliomas (**Table 1**).

### 3.2 CD44

CD44 is a transmembrane glycoprotein and a significant member of the cell adhesion molecule family, widely recognized as a cancer stem cell (CSC) marker (34). In addition to its roles in stemness and invasion, emerging studies indicate that CD44 may participate in the development of radioresistance by modulating DNA damage repair processes and hypoxia-driven signaling (35). CD44 has been implicated in the activation of downstream pathways involving AKT1 and DNA-PKcs, resulting in upregulation of critical non-homologous end-joining (NHEJ) repair proteins, such as DNA-PKcs, KU70, and KU80, and thereby enhancing the repair of radiation-induced DNA double-strand breaks (36). Moreover, CD44 expression is strongly associated with hypoxic tumor microenvironments in glioma. Hypoxia-induced activation of HIF-1α and stabilization of HIF-2α further promote CD44 expression, supporting hypoxia-adaptive gene programs linked to stemness and resistance to radiotherapy in perivascular niches (37). CD44 gene alterations is radioresistance markers for radiotherapy efficacy. Post-radiotherapy, the proportion of CD44-positive cells in tumors generally increases, further contributing to reduced therapeutic efficacy and heightened tumor recurrence (38). A retrospective clinical study demonstrate that patients with high CD44 expression in GBM have a significantly shortened median OS of 3.5 months compared to 18.5 months in low-expression patients (HR=3.216, p<0.004) (39). Elevated CD44 expression predicts poorer radiotherapy outcomes, indicating that CD44-targeted therapies may substantially improve patient prognosis (**Table 1**).

### 3.3 PRMT1

PRMT1 gene alterations significantly influence radiotherapy efficacy in glioma patients. Studies have revealed that PRMT1 overexpression or genetic variations are closely associated with radiotherapy resistance, particularly in glioma stem cells (GSCs) (40). By inhibiting ferroptosis and enhancing DNA damage repair capacity, tumors in patients with PRMT1 alterations exhibit heightened tolerance to radiotherapy (41). Specifically, tumor shrinkage rates in PRMT1-high patients were only 15%, compared to 45% in PRMT1-low patients (p < 0.01). Furthermore, the median progression-free survival (PFS) was 6.2 months for patients with high PRMT1 expression, while those with low expression achieved 12.3 months (p < 0.05) (41). Another study demonstrated that the incidence of pseudoprogression was significantly reduced in PRMT1-altered patients (8%) compared to non-mutated patients (27%) (42). These findings highlight PRMT1 genetic alterations as critical biomarkers for predicting radiotherapy outcomes in glioma patients, offering a foundation for developing personalized therapeutic strategies (**Table 1**).

### 3.4 CSF-1R

CSF-1R (Colony Stimulating Factor 1 Receptor) gene alterations significantly impact radiotherapy efficacy in glioma patients, primarily through their regulatory role on tumor-associated macrophages (TAMs) and immune responses within the tumor microenvironment (43). Studies have shown that CSF-1R mutations or overexpression enhance the pro-tumorigenic properties of TAMs, reducing sensitivity to radiotherapy. Conversely, inhibiting CSF-1R can reprogram TAMs, thereby improving radiotherapeutic outcomes (44). Data indicate that tumor control rates in patients with high CSF-1R expression were 42%, significantly lower than the 78% observed in patients with low expression (p < 0.01) (45). Moreover, combining radiotherapy with a CSF-1R inhibitor extended the median PFS to 14.8 months, compared to 7.5 months in patients receiving radiotherapy alone (p < 0.001) (45). Further demonstrated in a high-CSF-1R mouse model that tumor shrinkage rates were significantly lower compared to controls (35% vs. 65%, p < 0.05), reinforcing the critical role of CSF-1R (46). Collectively, these findings establish CSF-1R genetic alterations as key predictive biomarkers for radiotherapy efficacy in gliomas and suggest that targeted inhibition of CSF-1R could substantially enhance therapeutic outcomes. Despite these encouraging observations, the clinical translation of CSF-1R–targeted therapies has yielded heterogeneous outcomes. Several early-phase clinical studies have demonstrated only modest therapeutic benefit, which is frequently attributed to adaptive resistance mechanisms within the tumor microenvironment (47, 48). Accumulating mechanistic evidence indicates that CSF-1R blockade can lead to functional reprogramming of tumor-associated macrophages (TAMs), resulting in the secretion of compensatory growth factors, such as insulin-like growth factor-1 (IGF-1). In parallel, radiation-induced heterogeneity of the vascular niche and resident microglia may further attenuate the durability of immune activation anticipated from CSF-1R inhibition (49). Collectively, these findings suggest that although CSF-1R represents a relevant resistance-associated biomarker and therapeutic target, monotherapy is unlikely to achieve sustained clinical efficacy, thereby underscoring the rationale for combination strategies aimed at overcoming adaptive microenvironmental compensation (**Table 1**).

### 3.5 RAD51

RAD51 gene variations are pivotal in determining radiotherapy efficacy among glioblastoma (GBM) patients (50). The RAD51-encoded protein is integral to homologous recombination repair, ensuring genomic stability through precise DNA double-strand break repair (51). Dysregulated RAD51 expression is strongly associated with oncogenesis and genomic instability, often leading to diminished sensitivity to radiotherapy (52). Studies reveal that GBM patients with high RAD51 expression have a median overall survival (OS) of 10.6 months, significantly lower than the 20.1 months observed in those with low expression (p = 0.03). Additionally, multivariate Cox analysis demonstrates that elevated RAD51 levels substantially increase mortality risk (HR = 3.49, p = 0.03) (53). These results indicate that RAD51 serves as a crucial molecular biomarker for predicting poor radiotherapy outcomes in GBM patients. Furthermore, targeting RAD51 presents a promising strategy to enhance radiotherapeutic efficacy and improve patient prognosis (**Table 1**).

### 3.6 HMGB2

HMGB2 (High Mobility Group Box 2) gene alterations influence radiotherapy efficacy in glioma by promoting radioresistance through enhanced DNA repair mechanisms and immune response pathways (54, 55). Elevated HMGB2 expression has been implicated in the acquisition of radioresistance in glioma cells (56). Preclinical *in vitro* studies demonstrate that HMGB2 overexpression is associated with enhanced proliferative capacity following irradiation, with significantly higher proliferation rates observed at both 0 Gy and 6 Gy compared with control cells, whereas HMGB2 knockdown results in reduced proliferation and increased radioresistance (57). Notably, HMGB2 silencing markedly augments radiation-induced cell death, while HMGB2 overexpression attenuates apoptotic responses after 6 Gy exposure. Consistent with these phenotypic changes, radiobiological analyses reveal that HMGB2 depletion leads to decreased survival fraction at 2 Gy (SF2), reduced D0, Dq, and extrapolation number (N), accompanied by an increased sensitizer enhancement ratio (SER). In contrast, HMGB2 overexpression increases SF2, D0, Dq, and N values while reducing SER, collectively indicating a more radioresistant cellular phenotype (57) (**Table 1**).

## 4 Mechanisms associated with radioresistance or radiosensitivity

### 4.1 Radiosensitivity

#### 4.1.1 Metabolic reprogramming

IDH mutations are central to the radioresistance of gliomas, with their mechanisms primarily involving metabolic reprogramming and the inhibition of DNA repair, which together enhance tumor cell sensitivity to radiotherapy (58). The IDH mutation, particularly the IDH1R132H variant, alters cellular metabolic pathways, resulting in the accumulation of 2-hydroxyglutarate (2-HG), which in turn affects the cellular redox state and DNA repair efficiency (59). The accumulation of 2-HG suppresses the activity of intracellular antioxidant enzymes, increasing the cells’ susceptibility to oxidative damage during radiotherapy (60). Furthermore, IDH mutations downregulate DNA repair genes, such as TIGAR, inhibiting the DNA damage response and promoting the accumulation of radiation-induced DNA damage, thus enhancing the therapeutic effects of radiotherapy (61). Research indicates that glioma cells with IDH mutations exhibit higher levels of DNA damage and, due to compromised repair mechanisms, are more sensitive to radiotherapy (16). These findings suggest that IDH mutations could serve as a biomarker for glioma radiosensitivity and offer potential avenues for novel therapeutic interventions to address radioresistance (**Table 2**).

**Table 2:** Mechanisms associated with radioresistance or radiosensitivity.

Biomarker	Mechanisms	Pathways or tumor microenvironment features		Reference
IDH1 mutations MGMT promoter methylation TIM-3 CD133 CD44 PRMT1 CSF-1R RAD51 HMGB2	Metabolic reprogramming DNA damage repair Tumor Microenvironment Tumor Microenvironment Tumor Microenvironment Ferroptosis Tumor Microenvironment DNA damage repair DNA damage repair	/ / Impairs dendritic cell–mediated antigen presentation Activated ATM/ATR signaling ECM interaction and cancer stemness Regulated Nrf2 pathway M2 macrophage polarization Homologous recombination repair pathway /	(59) (64) (79, 80) (66) (83) (73) (84) (68) (69)	

#### 4.1.2 DNA damage repair

The methylation of the MGMT (O6-methylguanine-DNA methyltransferase) gene is fundamental to the radiotherapy sensitivity of gliomas. The protein encoded by the MGMT gene is vital for repairing DNA alkylation damage caused by radiotherapy. Methylation of MGMT leads to the silencing of its gene expression, impairing the tumor cells’ ability to repair alkylation-induced DNA damage, which in turn heightens their sensitivity to radiotherapy (62). Research has demonstrated that glioma cells with MGMT methylation show an enhanced response to radiotherapy, particularly when treated with alkylating agents like temozolomide, resulting in more effective treatment outcomes (63). MGMT methylation hinders the repair of DNA damage induced by radiotherapy, intensifying cell death and further increasing the sensitivity of tumor cells to radiation (21). Furthermore, the methylation status of MGMT is extensively utilized as a biomarker for predicting the radiosensitivity of gliomas, holding significant clinical prognostic value (64). The presence of MGMT methylation provides a new avenue for personalized radiotherapy, where targeting and inhibiting MGMT can improve the efficacy of radiation treatment, leading to better therapeutic results (**Table 2**).

### 4.2 Radioresistance

#### 4.2.1 DNA damage repair and the cell cycle

CD133, RAD51, and HMGB2 have been identified as important molecular determinants of radioresistance in glioblastoma, predominantly through their coordinated involvement in DNA damage repair and cell cycle regulation. CD133-positive glioma cells exhibit a markedly enhanced capacity to resolve radiotherapy-induced DNA double-strand breaks, a phenotype driven by activation of the ATM/ATR signaling cascade and efficient recruitment of DNA repair machinery, including RAD51 (65). In parallel, enrichment of CD133-positive cells is associated with sustained activation of the G2/M checkpoint, thereby prolonging the DNA repair window and promoting tumor cell survival following irradiation (31, 66). RAD51 itself represents a central and independent mediator of radioresistance. As a core component of the homologous recombination repair (HRR) pathway, RAD51 is frequently overexpressed in GBM, enabling efficient repair of radiation-induced DNA lesions and preservation of genomic stability (67). Beyond its canonical role in DNA repair, RAD51 also interacts with G2/M checkpoint regulation and anti-apoptotic signaling pathways, further suppressing radiation-induced cell death and facilitating tumor persistence and recurrence (68). In addition to these repair effectors, HMGB2 has emerged as an upstream chromatin-associated regulator that independently contributes to glioma radioresistance by modulating the DNA damage response. By binding to damaged DNA and maintaining an open chromatin configuration, HMGB2 facilitates the recruitment and assembly of repair complexes at irradiation-induced lesions (69). Accumulating evidence indicates that HMGB2 overexpression enhances DNA repair efficiency—particularly through activation of base excision repair–related pathways—reduces γ-H2AX accumulation, and attenuates radiation-induced apoptosis (56). Collectively, CD133, RAD51, and HMGB2 contribute to GBM radioresistance through distinct yet convergent mechanisms centered on enhanced DNA repair capacity and cell cycle control, highlighting these molecules as complementary therapeutic targets for radiosensitization strategies (**Table 2**).

#### 4.2.2 Ferroptosis

PRMT1 (protein arginine methyltransferase 1) is crucial in mediating the radioresistance of gliomas, primarily by suppressing ferroptosis, which shields tumor cells from the detrimental effects of radiotherapy (41). Ferroptosis is an iron-dependent form of cell death, marked by significant lipid peroxidation and elevated levels of reactive oxygen species (ROS) (70, 71). In gliomas, PRMT1 regulates several cellular processes through its methyltransferase activity, reducing ROS accumulation and inhibiting lipid peroxidation, thereby preventing ferroptosis (72). Research indicates that PRMT1 maintains intracellular iron homeostasis and diminishes iron-induced lipid peroxidation by modulating the antioxidant enzyme system, especially through the regulation of the Nrf2 pathway, ultimately blocking the onset of ferroptosis (73). Furthermore, PRMT1 prevents radiation-induced cell death by influencing fatty acid metabolism and stabilizing the cell membrane (74). In conclusion, PRMT1 alleviates the ferroptosis process induced by radiotherapy via multiple mechanisms, offering a promising therapeutic target for overcoming glioma radioresistance (**Table 2**).

### 4.3 Tumor microenvironment

The glioma tumor microenvironment (TME) critically shapes radiotherapy response through coordinated vascular, immune, and stromal adaptations (75, 76). Radiotherapy-induced vascular abnormalities and blood–brain barrier disruption exacerbate regional hypoxia and metabolic stress, thereby attenuating DNA double-strand break formation and activating hypoxia-dependent survival pathways (77). In parallel, irradiation triggers dynamic immune remodeling, characterized by a transient increase in antigen release and immune cell infiltration followed by compensatory immunosuppressive reprogramming that limits durable antitumor immunity (78). Within this context, TIM-3, CD44, and CSF-1R play distinct yet interconnected roles in modulating radiotherapy outcomes.TIM-3 is predominantly induced in response to irradiation and reflects radiation-driven immune dysfunction; its upregulation suppresses T-cell receptor signaling, impairs dendritic cell–mediated antigen presentation, and attenuates inflammasome activation, thereby dampening radiation-induced immunogenic cell death (79, 80). As such, TIM-3 functions as a radiosensitivity-associated marker, indicating the extent of radiotherapy-induced immune exhaustion and providing a rational target for restoring immune responsiveness (81). In contrast, CD44 and CSF-1R primarily operate as radioresistance-associated markers by reinforcing stromal and myeloid-mediated protective niches within the TME.CD44 engages in interactions with extracellular matrix components such as hyaluronic acid, thereby augmenting tumor cell adhesion and migratory capabilities (82). This interaction is intrinsically linked to the maintenance of cancer stem cell phenotypes, endowing cells with enhanced self-renewal and anti-apoptotic properties. Furthermore, CD44 orchestrates the regulation of inflammatory signaling pathways, fostering an immunosuppressive milieu within the TME that facilitates tumor evasion from immune surveillance (83). Conversely, CSF-1R predominantly influences the polarization of tumor-associated macrophages (TAMs) towards the immunosuppressive M2 phenotype (46, 84). This polarization is characterized by the secretion of pro-tumorigenic cytokines such as TGF-β and IL-10, which collectively support tumor cell survival and bolster resistance to radiotherapeutic interventions (85). The M2-polarized TAMs contribute to an environment that not only promotes tumor growth but also impairs the efficacy of radiotherapy by enhancing the reparative and survival mechanisms of cancer cells. Empirical studies have demonstrated that targeted inhibition of CD44 and CSF-1R can effectively remodel the TME, thereby enhancing the sensitivity of GBM to radiotherapy (86). By disrupting the adhesive and migratory functions of CD44, as well as altering the cytokine milieu through CSF-1R antagonism, these therapeutic strategies mitigate the protective niches that facilitate tumor resistance (87). Consequently, the simultaneous targeting of CD44 and CSF-1R emerges as a promising approach to overcome radiotherapy resistance, offering significant therapeutic potential for improving clinical outcomes in patients with GBM (43, 88). In summary, CD44 and CSF-1R are instrumental in shaping a TME that promotes radiotherapy resistance in GBM. Their roles in enhancing tumor cell adhesion, migration, stemness, and immunosuppression underscore their importance as therapeutic targets (89). Collectively, these pathways converge to establish a radiation-adaptive microenvironment in which TIM-3 reflects radiotherapy-associated immune sensitivity, whereas CD44 and CSF-1R actively drive adaptive resistance, underscoring the rationale for biomarker-guided combination strategies to overcome TME-mediated radiotherapy failure in glioma (**Table 2**).

## 5 Value of personalized radiotherapy

Biomarker-guided precision radiotherapy is gaining increasing attention in neuro-oncology and reflects a shift toward individualized treatment for gliomas. Molecular biomarkers are now part of routine clinical decision-making and are used to stratify patients and adjust radiotherapy based on predicted treatment response. MGMT promoter methylation is a key biomarker for radiotherapy optimization in glioblastoma. Patients with MGMT promoter methylation show increased sensitivity to temozolomide and can receive reduced radiation doses. In this group, lowering the dose from 60 Gy to 52–54 Gy is linked to improved survival, with a median overall survival of 32 months compared with 25 months (90). This benefit is seen in patients with gross total resection and subtotal resection. Patients with unmethylated MGMT promoters show limited chemosensitivity and still require the standard dose of 60 Gy. This effect is most clear after subtotal resection, where dose reduction leads to worse outcomes, with median overall survival decreasing from 11 months to 8 months (p = 0.011), indicating a high risk of undertreatment in this group (90). The phase III SPECTRO-GLIO trial shows the value of molecular stratification for radiotherapy dose escalation. In this study, metabolic imaging–guided escalation to tumor regions up to 72 Gy combined with temozolomide did not improve outcomes in the overall cohort compared with standard chemoradiotherapy, with median overall survival of 22.6 versus 22.2 months and median progression-free survival of 8.6 versus 7.8 months (91). Patients with MGMT promoter methylation showed improved survival after dose escalation, with a median overall survival of 38.0 months compared with 28.5 months (91). These results show that MGMT promoter methylation guides both radiotherapy dose reduction and dose escalation and supports biomarker-based patient selection to improve benefit and reduce toxicity. Immune-related biomarkers also guide treatment selection.Also,TIM-3 expression reflects radiotherapy-induced immune changes. Standard 6 Gy irradiation increases TIM-3 expression by two- to threefold in tumor cells and immune cells in the tumor microenvironment. In NP53 preclinical models, tumors with high TIM-3 expression show better survival after combined radiotherapy and TIM-3 inhibition, with median survival of 58 versus 30 days, and about half of treated subjects achieve long-term survival (27). Tumors with low TIM-3 expression respond better to standard radiotherapy or other immune checkpoint approaches. Despite these promising advances, Biomarker-based radiotherapy is still early in clinical use. Future studies should focus on multicenter trials to confirm dose–response relationships and to build translational frameworks that combine biological mechanisms with precision radiotherapy.

Multi-omics approaches and artificial intelligence further advance precision radiotherapy beyond single-gene models. Combined genomic, transcriptomic, proteomic, and radiomic analyses allow detailed evaluation of tumor heterogeneity and biomarker changes over time. Multigene expression models that include the AR/cJun/STAT1/PKC signaling axis and radiosensitivity indices stratify glioblastoma by radioresistance. Clinical studies show that patients with high RSI treated with chemoradiation have shorter overall survival, with a hazard ratio of 1.64 (p = 0.02). This effect is strongest in MGMT-overexpressing subgroups, where 1-year survival is 84.1% in radiosensitive patients and 53.7% in radioresistant patients (p = 0.005) (92). RSI acts as an independent prognostic biomarker and supports treatment decisions in MGMT-overexpressing patients.AI-driven multimodal prognostic architectures demonstrate superior clinical efficacy. In one cohort study, a support vector machine combining radiomic, dosiomic, and clinical features was trained on 176 patients and reached an AUC of 0.728 in the validation cohort (95% CI: 0.717–0.739), which was higher than single-modality models (93). Dosiomic features contributed most to model accuracy and suggest that spatial dose heterogeneity reflects tumor radiosensitivity or resistance. These results support using dose distribution information in personalized radiotherapy planning. But Clinical translation of AI-guided radiotherapy in glioma is still limited, Most current models use small retrospective single-center datasets and show limited generalizability across centers with different imaging protocols and workflows. Differences in image acquisition, target delineation, and dose calculation reduce feature reproducibility. Limited model interpretability lowers clinician confidence and complicates regulatory evaluation. Progress will require prospective multicenter validation, standardized data collection and reporting, and explainable AI frameworks to support safe clinical use.

## 6 Conclusion

Radiotherapy remains a cornerstone of glioma treatment; however, the intrinsic radiation resistance and heterogeneity of glioma continue to impede favorable clinical outcomes. The identification and integration of molecular markers into clinical practice represent a transformative approach to addressing these challenges. This review emphasizes the crucial roles of both positive and negative radiotherapy efficacy markers in optimizing the management of glioma radiotherapy.

Molecular biomarkers provide dual guidance for precision radiotherapy in gliomas through synergistic integration of prognostic and predictive functionalities. Prognostic biomarkers, exemplified by IDH1 mutations and TERT promoter alterations, reflect inherent tumor biological properties through mechanisms indirectly associated with radiosensitivity. For instance, IDH1 mutations induce 2-hydroxyglutarate accumulation that impairs DNA repair pathways, though their prognostic significance remains independent of therapeutic regimens. Predictive biomarkers such as MGMT promoter methylation and TIM-3 overexpression directly modulate therapeutic responses: MGMT hypermethylation enhances chemoradiation synergy via epigenetic silencing of DNA repair enzymes, while TIM-3 upregulation mediates radioresistance through immunosuppressive microenvironment remodeling via CD8 T-cell exclusion and NLRP3 inflammasome suppression, with pharmacological inhibition of which potentiating PD-1 inhibitor efficacy. Radioresistance biomarkers including CD133 and RAD51 exhibit dual functionality. CD133 sustains stemness through ATM/ATR pathway activation and DNA damage response potentiation, thereby exacerbating locoregional recurrence risks.RAD51 overexpression compromises radiosensitivity via homologous recombination repair (HRR)-mediated double-strand break resolution. In addition, HMGB2 contributes to radioresistance by preserving an open chromatin architecture, which facilitates the efficient recruitment and assembly of DNA repair complexes at irradiation-induced DNA lesions, ultimately enhancing post-irradiation DNA damage repair capacity. This mechanism-driven tripartite classification system optimizes clinical paradigms: prognostic stratification informs risk assessment, predictive signatures guide therapeutic personalization, while resistance markers provide rationale for combinatorial approaches integrating radiotherapy with targeted molecular interventions.

The synergistic convergence of molecular biomarkers and artificial intelligence (AI) technologies is ushering in an unprecedented era of personalized radiotherapy. Tumor radiosensitivity emerges as a multidimensional phenotype orchestrated by molecular determinants spanning DNA repair fidelity, immunomodulatory networks, stemness maintenance programs, and hypoxic signaling cascades (94). Established predictive paradigms including Radiosensitivity Index (RSI) and AI machine learning models have demonstrated quantifiable prognostic capabilities through integrative analysis of transcriptomic datasets and radiobiological parameters. Building upon these foundations, AI-driven multidimensional frameworks incorporating genomic aberrations, radiomic signatures, and clinical covariates achieve enhanced predictive precision. Genetic lesions within DNA repair pathways (e.g., ATM/BRCA1 mutations) inform radiotherapy optimization strategies, whereas hypoxia-responsive gene signatures enable identification of candidates for hypoxia-targeted adjunctive therapies (95). Currently, select validated models have transitioned into clinical implementation, while emerging prototypes remain under rigorous translational validation. Although prospective multicenter trials are warranted, extant evidence substantiates their utility in dosimetric personalization and patient stratification (96). This evolutionary trajectory portends a transformative paradigm shift—precision multimodal integration of radiotherapy with molecularly targeted agents and immunotherapeutics, thus delineating novel therapeutic frontiers in glioma management.

In summary, the integration of molecular markers into radiotherapy paradigms marks a paradigm shift toward precision oncology in glioma management. By decoding the molecular basis of radioresistance and leveraging biomarkers for therapeutic innovation, we can transform radiotherapy from a generic intervention into a precision tool, offering renewed hope for patients with this devastating disease.
